# Development and characterization of polymorphic microsatellite loci for spiny-footed lizards, *Acanthodactylusscutellatus* group (Reptilia, Lacertidae) from arid regions

**DOI:** 10.1186/s13104-015-1779-3

**Published:** 2015-12-17

**Authors:** Sara Cristina Lopes, Guillermo Velo-Antón, Paulo Pereira, Susana Lopes, Raquel Godinho, Pierre-André Crochet, José Carlos Brito

**Affiliations:** CIBIO/InBIO, Centro de Investigação em Biodiversidade e Recursos Genéticos, Universidade do Porto. Campus Agrário de Vairão, 4485-661 Vairão, Portugal; Departamento de Biologia, Faculdade de Ciências, Universidade do Porto. Rua Campo Alegre s/n, 4169-007 Porto, Portugal; CNRS-UMR 5175, Centre d’Ecologie Fonctionnelle et Evolutive, 1919 route de Mende, 34293 Montpellier-Cedex 5, France

**Keywords:** Cross-species amplification, Nuclear markers, Population genetics, Sahara-Sahel

## Abstract

**Background:**

Spiny-footed lizards constitute a diverse but scarcely studied genus. Microsatellite markers would help increasing the knowledge about species boundaries, patterns of genetic diversity and structure, and gene flow dynamics. We developed a set of 22 polymorphic microsatellite loci for cross-species amplification in three taxa belonging to the *Acanthodactylus scutellatus* species group, *A. aureus*, *A. dumerili/A. senegalensis* and *A. longipes*, and tested the same markers in two other members of the group, *A. scutellatus* and *A. taghitensis*.

**Results:**

Amplifications in *A. aureus*, *A. longipes* and *A. dumerili*/*A.senegalensis* were successful, with markers exhibiting a number of alleles varying between 1 and 19. Expected and observed heterozygosity ranged, respectively, between 0.046–0.893 and 0.048–1.000. Moreover, 17 and 16 loci were successfully amplified in *A. scutellatus* and *A. taghitensis*, respectively.

**Conclusion:**

These markers are provided as reliable genetic tools to use in future evolutionary, behavioural and conservation studies involving species from the *A. scutellatus* group.

## Background

Spiny-footed lizards, or fringe-toed lizards (genus *Acanthodactylus*), form a clade of small ground-dwelling lizards occurring mostly in arid regions [1, 2]. The genus is the most specious of the Lacertidae family and is widely distributed, occurring from the Iberian Peninsula, south of the Mediterranean Basin, across the Sahara-Sahel, Arabian Peninsula, and as far east as India [1, 2]. Being often abundant and occupying different types of open, flat habitats, these lizards are important elements of the vertebrate communities of deserts and arid ecosystems in North Africa and Arabia. Despite their diversity, knowledge about most of the species is still scarce and their taxonomy is partly unresolved [1–4]. Most authors agree on splitting *Acanthodactylus* into several species groups or complexes [1, 4]. The *A. scutellatus* species group shows one of most complex taxonomies [2, 5–8]. It includes six species according to the last global revision (*A. aureus, A. dumerili, A. longipes, A. scutellatus, A. senegalensis* and *A. taghitensi*s) [3]. However, urgent systematic revision based on molecular data is needed given that: (1) eastern populations previously attributed to *A. longipes* are now considered a new species (*A. aegyptius*, [7]); and (2) species boundaries in *A. scutellatus*, *A. longipes*, *A. dumerili* and *A. senegalensis* as currently defined remain uncertain (own unpublished data, SC Lopes, Velo-Antón, Crochet, Brito). The species group has multiple forms occurring in sympatry in Mauritania—*A. aureus*, *A. dumerili, A. senegalensis,* and *A. longipes* [9]. In this contact zone, morphologically intermediate individuals were previously observed [3] and molecular studies are needed to distinguish whether high morphological diversity or hybridization explain these intermediate morphotypes. In addition, assessment of gene flow in such areas of sympatry would be critical for a better understanding of the species boundaries. Microsatellite markers have been extremely useful, and affordable, for addressing numerous topics in conservation and evolutionary biology, allowing, e.g., gene flow and population structure assessments, demographic inferences and genetic diversity estimation [10–12]. Yet, no microsatellite markers are available for the *Acanthodactylus* genus.

Here we describe a set of 22 polymorphic microsatellite loci (tri- and tetranucleotides) characterized in four species included in the *A. scutellatus* species group (*A. aureus*, *A. longipes* and *A. dumerili*/*A.senegalensis*). Considering the uncertain species boundaries for *A. dumerili* and *A. senegalensis*, we chose to refer to them as *A. dumerili/A. senegalensis* in the following sections. We further tested cross-amplification of these markers in two other members of the species group, *A. scutellatus* and *A. taghitensis*.

## Methods

A genomic library was constructed from 12 specimens of *A. aureus*, collected across the species’ distribution. A tissue sample was collected from the tail tip by following ethical guidelines for use of live reptiles (http://www.aaalac.org/accreditation/Guidelines_for_Use_of_Live_Amphibians_and_Reptiles.pdf). All specimens were released on site after sample collection. Fieldwork was developed with permission from the Ministére Délégué auprès du Premier Ministre Chargé de l’Environnement, Nouakchott (Permit: 460/MDE/PNBA) and from the Haut Commissariat aux Eaux et Forêts et à la Lutte Contre la Désertification, Rabat (Permits 256-2012 and 20-2013). Analyses were done at a CITES registered laboratory: 13PT0065/S. Field collection and handling practices were approved by the Committee of Animal Experimentation of the University of Porto (Portugal) under the Directive 2010/63/EU of the European Parliament.

**Fig. 1:**
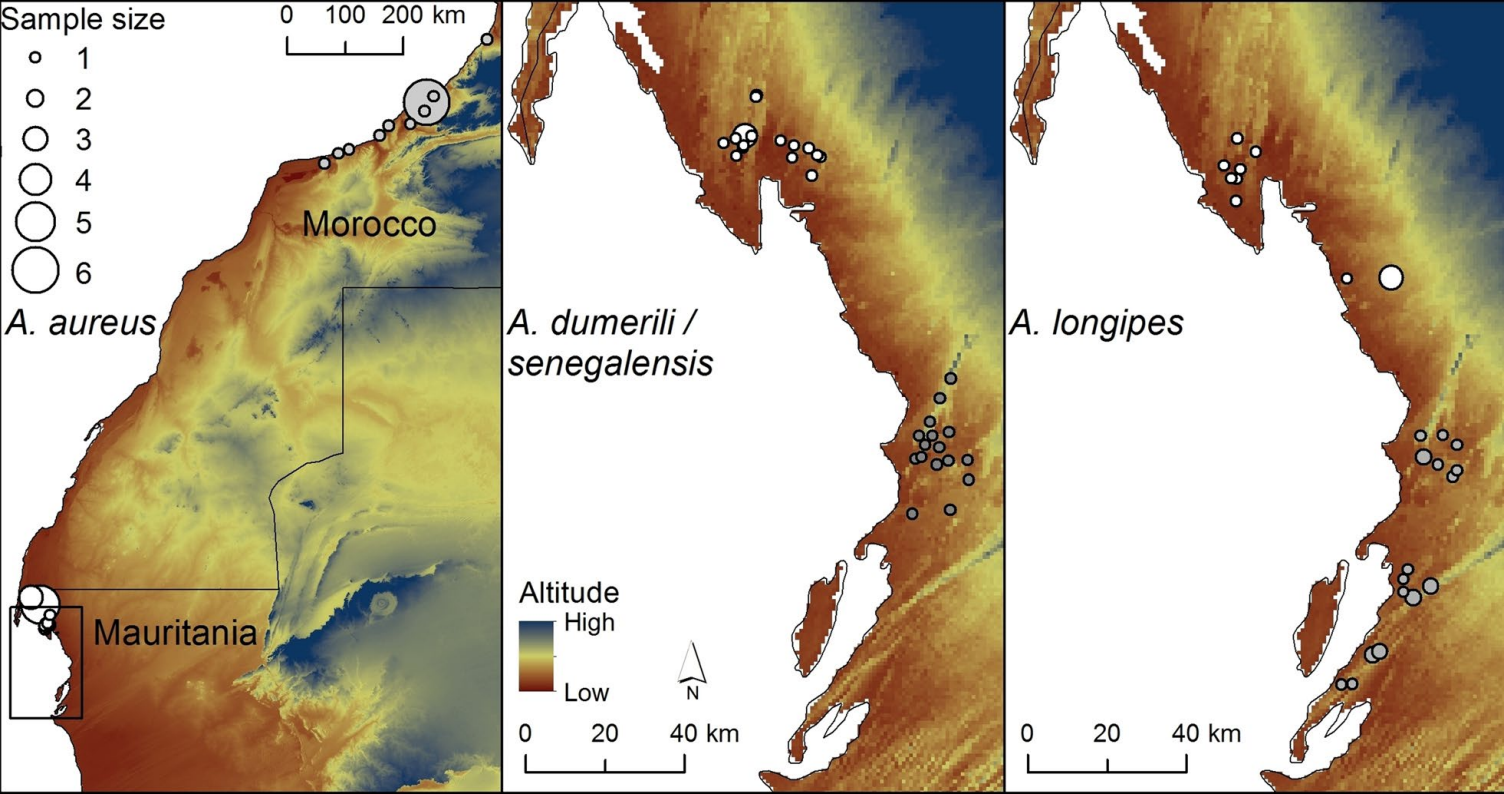
Distribution of genotyped samples used for *Acanthodactylus aureus, A. dumerili/senegalensis* and *A. longipes*. *White circles* correspond to Pop1, while *grey* *circles* correspond to Pop2. *Circles* are proportional to sample size. The *rectangle* in the map of *A. aureus* represents the area depicted in the maps of *A. dumerili/senegalensis* and *A. longipes.* The samples sizes of the populations are the following: Pop1 = 21 and Pop2 = 17 in *A. aureus*; Pop1 = 24 and Pop2 = 19 in *A. dumerili/A. senegalensis*; and Pop1 = 14 and Pop2 = 19 in *A. longipes*

Genomic DNA extractions were performed from tissue samples using EasySpin Kit (Qiagen), following an adapted protocol for tissue samples (with minor adjustments to centrifugation and incubation conditions) and then pooled in equimolar concentrations. The changes to the extraction protocol were as follows: after adding the AB solution, we centrifuged at 3700 rpm for 4 min (instead of 4000 rpm for 2 min). After adding the Wash solution, we centrifuged at 3700 rpm for 6 min (instead of 8000 rpm for 1 min). After repeating the Wash solution step and discarding flow-through, we centrifuged at 3700 rpm for 10 min (instead of 14,000 rpm for 5 min). After adding the Elution Buffer, we incubated at 55° for 15 min (instead of 50° for 10 min). Last centrifugation was at 3700 rpm (instead of 14,000 rpm). Microsatellite isolation was developed through 454 GS-FLX Titanium pyrosequencing of enriched DNA libraries [13]. This process was developed by GenoScreen (http://www.pasteur-lille.fr/fr/recherche/plateformes/tordeux_plat.html) and included sequence data quality control, assembly and analyses, and primer design. Initially, 50 loci were selected from the library and tested for amplification using seven samples of *A. aureus*, *A. dumerili/A. senegalensis*, and *A. longipes*. Thirty loci amplified reliably, producing fragments of the expected size. Twenty-two were polymorphic (Table 1), and amplified with differential success in the following target species: 21 in *A. aureus*, 18 in *A. longipes* and 15 in *A. dumerili*/*A.senegalensis*. These 22 loci were therefore used for genotyping 38 samples of *A. aureus*, 35 of *A. longipes*, and 43 of *A. dumerili/A. senegalensis*, collected along coastal Morocco and Mauritania (Table 2; Fig. 1). Markers were multiplexed in four reactions, using M13-primer genotyping protocol with four different dye-labelled tails, and forward primer concentration of 1/10 of dye-labelled reverse primer [14] (Table 1). The transferability of the primers was tested by cross-amplification of five specimens of *A. scutellatus* (from Morocco, Tunisia, Libya, Algeria and Egypt) and one specimen of *A. taghitensis* (Mauritania). PCR amplifications were conducted using the Multiplex PCR Kit (QIAGEN) following manufacturer’s instructions in a final 10 μl volume, always in the presence of a negative control. Touchdown PCR conditions started with an initial denaturation step of 15 min at 95 °C; first round (nine cycles) of 30 s at 95 °C, 90 s for annealing (decreasing 0.5 °C per cycle) at 58–54 °C (Multiplexes 1, 2 and 3) or 55–51 °C (Multiplex 4), and 30 s at 72 °C; second round (31 cycles) of 30 s at 95 °C, 1 min at 54 °C (Multiplexes 1, 2 and 3), or 51 °C (Multiplex 4), 30 s at 72 °C, and a final extension of 30 min at 60 °C. Amplification was performed in Biorad T100 Thermal Cyclers, and the PCR products were later separated by capillary electrophoresis on an automatic sequencer *ABI3130xl Genetic Analyzer* (AB Applied Biosystems). Fragments were scored against the GeneScan-500 LIZ Size Standard using the GENEMAPPER 4.1 (Applied Biosystems) and manually checked twice. Potential evidences of null alleles, allelic dropouts and stuttering were assessed using MICRO-CHECKER v2.2.3 [15] at each locus, for each population. Tests for Hardy–Weinberg equilibrium (HWE) and linkage disequilibrium (LD) were assessed in GENEPOP online version (http://wbiomed.curtin.edu.au/genepop/); with subsequent Bonferroni correction in both cases. Observed and expected heterozygosity were computed using GenAlEx v6.501 [16]. For some populations, samples were obtained from different localities. Consequently, analyses were based on groups of samples that are not necessarily panmitic populations, which probably accounts for deviations from Hardy–Weinberg equilibrium.

**Table 1:** Global characterization of the 22 microsatellite loci characterized in *Acanthodactylus aureus*, *A. dumerili/A. senegalensis* and *A. longipes*

Locus	GenBank assess no.	Repeat	Primer sequence (5′–3′)	Multiplex	TD	Dye
Ac1	KU295182	(ATAC)_8_	F: CTGTGGTATATCCCCTGCCA R: GGTGGCTTCTCCACAGCTATT	1	58°/54°	FAM
Ac4	KU295183	(TTC)_21_	F: ACAGCTCTGCAGTAATTCCATTT R: CCGATGCAGTGTTTCGTAGG	3	58°/54°	VIC
Ac5	KU295184	(AAC)_15_	F: GTTGCTTCAACTGCTCCTCC R: AGTGTCCTGTGCACAACCAG	1	58°/54º	VIC
Ac6	KU295185	(TTG)_10_	F: GTAGCCCAGTCAGATGGGTG R: CCTCCAACATTCCAGTCCAG	4	55°/51°	NED
Ac8	KU295186	(TTG)_11_	F: GACATCTGAAGGCAGCCCTA R: GGTTGTAGCCTGGAGCAGAA	1	58°/54°	NED
Ac9	KU295187	(CAA)_15_	F: TCATACAGGGATGTTTCAGGG R: GCAGGAGGAAGGAAGCTTTT	1	58°/54°	PET
Ac13	KU295188	(AAC)_14_	F: TCCATGGGGTCACAAAGAGT R: TCTCCAGCACTTATCTGATGC	2	58°/54°	FAM
Ac14	KU295189	(CAA)_10_	F: TTAAGTGGCAATGTGTTGCAT R: TCCCACATGGTGGGTTACTT	2	58°/54°	VIC
Ac16	KU295190	(AGG)_10_	F: AGTCAATTTATTCAAATGATCTTCCA R: TCATCCAAGAAAATCTGCTGC	2	58°/54°	VIC
Ac19	KU295191	(AAC)_14_	F: TCATTTCACTTCAAACCTGTGG R: ACTGATGTTGGGTTTGGAGC	2	58°/54°	PET
Ac20	KU295192	(GTT)_11_	F: ATGCATAAGTACGAAAAGGGGA R: TCTACAGAGAAAGAGAAATAACAACAA	2	58°/54°	PET
Ac23	KU295193	(CAT)_8_	F: GCGAACATGCACAAGGTTT R: ACCCTGCTTGGTTCTCATTG	1	58°/54°	FAM
Ac28	KU295194	(ACAT)_8_	F: TGTCCGAAATAGGATGGAGC R: GGAAAGCCAATGCCTCTACA	4	55°/51°	PET
Ac31	KU295195	(GTT)_10_	F: GAAGGGTTACAACTGCCTGG R: CAGTGCTTCAGCAACAGGAG	4	55°/51°	FAM
Ac32	KU295196	(TTC)_15_	F: TAGTCCGTAAACTTGTGGGTCA R: TTCTCAGACAACAGACACCCA	3	58°/54°	FAM
Ac33	KU295197	(TGT)_16_	F: GGCACTGAAATATGTGGTTTTG R: TGACATGCTTCGGTGAAGTC	3	58°/54°	FAM
Ac36	KU295198	(TGT)_9_	F: GTCACGTTGATTGCATTGCT R: GCCAACTGGGAAACCTAGC	3	58°/54°	VIC
Ac43	KU295199	(CAA)_13_	F: AGCTTTTGTACGTTCCTTTGC R: CCAGAGAAACACATATGCAAGC	4	55°/51°	FAM
Ac44	KU295200	(GGA)_11_	F: TCCTTAAGAAAGGTACTTAATGCCA R: TCTTTACGTAGTCCCTTTGTGG	4	55°/51°	VIC
Ac45	KU295201	(CAA)_10_	F: AGGCAATGGAAGACAGGGA R: GCCTACAGTTTGTGCATAGGG	4	55°/51°	VIC
Ac47	KU295202	(ACA)_11_	F: CTTGCCTCTTCGCTTTCTGT R: TCCGGACAGCATTCCTCTAC	4	55°/51°	NED
Ac49	KU295203	(AAC)_11_	F: CAAAGAAAATTGTTGGAGGGG R: GTAAAACATCGGAAGGCAGC	4	55°/51°	PET

*TD* touchdown temperatures

**Table 2:** Data on sampling localities for each species

Code	Species	Latitude	Longitude	Local	Country
6477	*A. aureus*	20.9444	−16.5494	Kerekchet et Teintâne, extreme N	Mauritania
A366	*A. aureus*	21.2182	−16.8432	Nouâdhibou, 40 km S of	Mauritania
A367	*A. aureus*	21.2182	−16.8432	Nouâdhibou, 40 km S of	Mauritania
A368	*A. aureus*	21.2182	−16.8432	Nouâdhibou, 40 km S of	Mauritania
A369	*A. aureus*	21.2182	−16.8432	Nouâdhibou, 40 km S of	Mauritania
A358	*A. aureus*	21.0978	−16.6998	Nouâdhibou, 70 km S of	Mauritania
A359	*A. aureus*	21.0978	−16.6998	Nouâdhibou, 70 km S of	Mauritania
A360	*A. aureus*	21.0978	−16.6998	Nouâdhibou, 70 km S of	Mauritania
A361	*A. aureus*	21.0978	−16.6998	Nouâdhibou, 70 km S of	Mauritania
A362	*A. aureus*	21.0978	−16.6998	Nouâdhibou, 70 km S of	Mauritania
A363	*A. aureus*	21.0978	−16.6998	Nouâdhibou, 70 km S of	Mauritania
6449	*A. aureus*	20.8233	−16.5882	PNBA: Kerekchet et Teintâne, central	Mauritania
6458	*A. aureus*	20.8023	−16.5718	PNBA: Kerekchet et Teintâne, central	Mauritania
5171	*A. aureus*	20.7190	−16.6195	PNBA: Kerekchet et Teintâne, central 2	Mauritania
5172	*A. aureus*	20.7190	−16.6195	PNBA: Kerekchet et Teintâne, central 2	Mauritania
5173	*A. aureus*	20.7251	−16.6291	PNBA: Kerekchet et Teintâne, W side 1	Mauritania
5176	*A. aureus*	20.7620	−16.6183	PNBA: Kerekchet et Teintâne, W side 3	Mauritania
6443	*A. aureus*	20.7764	−16.6287	PNBA: Kerekchet et Teintâne, Western face	Mauritania
6446	*A. aureus*	20.8115	−16.6158	PNBA: Kerekchet et Teintâne, Western face	Mauritania
6448	*A. aureus*	20.8115	−16.6158	PNBA: Kerekchet et Teintâne, Western face	Mauritania
6435	*A. aureus*	20.7938	−16.5462	PNBA: Sebkhet Dbâdeb et Teintâne, W margin	Mauritania
A437	*A. aureus*	28.8731	−10.7027	Aoreora, 15 km E of (Plage Blanche)	Morocco
A438	*A. aureus*	28.8731	−10.7027	Aoreora, 15 km E of (Plage Blanche)	Morocco
A439	*A. aureus*	28.8731	−10.7027	Aoreora, 15 km E of (Plage Blanche)	Morocco
A440	*A. aureus*	28.8731	−10.7027	Aoreora, 15 km E of (Plage Blanche)	Morocco
A441	*A. aureus*	28.8731	−10.7027	Aoreora, 15 km E of (Plage Blanche)	Morocco
A442	*A. aureus*	28.8731	−10.7027	Aoreora, 15 km E of (Plage Blanche)	Morocco
A443	*A. aureus*	28.8731	−10.7027	Aoreora, 15 km E of (Plage Blanche)	Morocco
A435	*A. aureus*	28.7447	−10.7438	Aoreora, 25 km S of	Morocco
A436	*A. aureus*	28.7447	−10.7438	Aoreora, 25 km S of	Morocco
A556	*A. aureus*	29.8511	−9.7706	Bou Soun	Morocco
10,625	*A. aureus*	28.5177	−11.2970	Douira, N of	Morocco
10,638	*A. aureus*	28.3701	−11.4387	Douira, S of	Morocco
10,634	*A. aureus*	28.1544	−11.9117	Laareig	Morocco
10,636	*A. aureus*	27.9291	−12.2945	Leirane	Morocco
9048	*A. aureus*	28.9662	−10.6000	Plage Blanche	Morocco
10,635	*A. aureus*	28.0875	−12.0814	Sidi Akhfennir	Morocco
10,624	*A. aureus*	28.5479	−10.9583	Tafnidilt	Morocco
6470	*A. dum./sen.*	20.9172	−16.5418	Kerekchet et Teintâne, extreme N	Mauritania
6473	*A. dum./sen.*	20.9204	−16.5415	Kerekchet et Teintâne, extreme N	Mauritania
6474	*A. dum./sen.*	20.9204	−16.5415	Kerekchet et Teintâne, extreme N	Mauritania
3618	*A. dum./sen.*	20.0500	−16.0582	PNBA: Adeim el Marrâr	Mauritania
5111	*A. dum./sen.*	19.9733	−16.1874	PNBA: Agreigrât, 1 km E of	Mauritania
6384	*A. dum./sen.*	20.1010	−16.1655	PNBA: Aguilâl	Mauritania
5126	*A. dum./sen.*	20.1287	−16.1581	PNBA: Aguilâl 1	Mauritania
5135	*A. dum./sen.*	20.1497	−16.1420	PNBA: Aguilâl 4	Mauritania
5120	*A. dum./sen.*	20.1498	−16.1719	PNBA: Aguilâl, 1 km W of	Mauritania
5158	*A. dum./sen.*	20.7802	−16.3944	PNBA: Amgheououas es Sâhli	Mauritania
5160	*A. dum./sen.*	20.7843	−16.4027	PNBA: Amgheououas es Sâhli	Mauritania
5162	*A. dum./sen.*	20.8007	−16.4227	PNBA: Amgheououas es Sâhli, 3 km NW of	Mauritania
6390	*A. dum./sen.*	20.1808	−16.1474	PNBA: Dlo’ Matai	Mauritania
6391	*A. dum./sen.*	20.1808	−16.1474	PNBA: Dlo’ Matai	Mauritania
6394	*A. dum./sen.*	20.2330	−16.1247	PNBA: Dlo’ Matai	Mauritania
2750	*A. dum./sen.*	20.2789	−16.1003	PNBA: Dló Matai	Mauritania
3622	*A. dum./sen.*	20.0934	−16.0613	PNBA: Grâret Zra	Mauritania
2768	*A. dum./sen.*	20.8070	−16.5701	PNBA: Kerekchet et Teintâne	Mauritania
2769	*A. dum./sen.*	20.8070	−16.5701	PNBA: Kerekchet et Teintâne	Mauritania
6450	*A. dum./sen.*	20.8233	−16.5882	PNBA: Kerekchet et Teintâne, central	Mauritania
6453	*A. dum./sen.*	20.8233	−16.5882	PNBA: Kerekchet et Teintâne, central	Mauritania
6456	*A. dum./sen.*	20.8023	−16.5718	PNBA: Kerekchet et Teintâne, central	Mauritania
6457	*A. dum./sen.*	20.8023	−16.5718	PNBA: Kerekchet et Teintâne, central	Mauritania
6460	*A. dum./sen.*	20.8283	−16.5672	PNBA: Kerekchet et Teintâne, central	Mauritania
6461	*A. dum./sen.*	20.8283	−16.5672	PNBA: Kerekchet et Teintâne, central	Mauritania
6462	*A. dum./sen.*	20.8283	−16.5672	PNBA: Kerekchet et Teintâne, central	Mauritania
6463	*A. dum./sen.*	20.8283	−16.5672	PNBA: Kerekchet et Teintâne, central	Mauritania
6468	*A. dum./sen.*	20.8294	−16.5518	PNBA: Kerekchet et Teintâne, central	Mauritania
6469	*A. dum./sen.*	20.8294	−16.5518	PNBA: Kerekchet et Teintâne, central	Mauritania
5181	*A. dum./sen.*	20.7831	−16.5865	PNBA: Kerekchet et Teintâne, central 3	Mauritania
6445	*A. dum./sen.*	20.8115	−16.6158	PNBA: Kerekchet et Teintâne, Western face	Mauritania
2763	*A. dum./sen.*	20.8060	−16.4561	PNBA: N of Baie d’Arguin	Mauritania
2743	*A. dum./sen.*	20.0964	−16.1798	PNBA: NE of El Mounâne	Mauritania
6377	*A. dum./sen.*	20.1233	−16.1266	PNBA: Oued Nouafferd	Mauritania
5139	*A. dum./sen.*	20.1574	−16.1037	PNBA: Oued Nouafferd 3	Mauritania
6375	*A. dum./sen.*	20.0845	−16.1313	PNBA: Oued Nouafferd, 2 km S of	Mauritania
6376	*A. dum./sen.*	20.0845	−16.1313	PNBA: Oued Nouafferd, 2 km S of	Mauritania
3615	*A. dum./sen.*	20.0928	−16.1059	PNBA: Râs Tafarît, 16 km E of	Mauritania
6433	*A. dum./sen.*	20.8173	−16.4858	PNBA: Sebkhet Dbâdeb et Teintâne, 2 km E of	Mauritania
6431	*A. dum./sen.*	20.7791	−16.4602	PNBA: Sebkhet Dbâdeb et Teintâne, 4 km E of	Mauritania
6426	*A. dum./sen.*	20.7395	−16.4150	PNBA: Sebkhet Dbâdeb et Teintâne, 8 km SE of	Mauritania
6363	*A. dum./sen.*	19.9808	−16.1016	PNBA: Taguîlâlet Jreik, 2 km W of	Mauritania
6364	*A. dum./sen.*	19.9808	−16.1016	PNBA: Taguîlâlet Jreik, 2 km W of	Mauritania
2745	*A. longipes*	20.0699	−16.0896	PNBA: 5 km E of El Mounâne	Mauritania
6319	*A. longipes*	19.6589	−16.2639	PNBA: Ackenjeîl	Mauritania
6320	*A. longipes*	19.6589	−16.2639	PNBA: Ackenjeîl	Mauritania
6369	*A. longipes*	20.0567	−16.0993	PNBA: Adeim el Marrâr, 4 km W of	Mauritania
6383	*A. longipes*	20.1010	−16.1655	PNBA: Aguilâl	Mauritania
6386	*A. longipes*	20.1010	−16.1655	PNBA: Aguilâl	Mauritania
5119	*A. longipes*	20.1498	−16.1719	PNBA: Aguilâl, 1 km W of	Mauritania
A344	*A. longipes*	20.5080	−16.2380	PNBA: Bir el Gareb, 15 km S of	Mauritania
A345	*A. longipes*	20.5080	−16.2380	PNBA: Bir el Gareb, 15 km S of	Mauritania
A346	*A. longipes*	20.5080	−16.2380	PNBA: Bir el Gareb, 15 km S of	Mauritania
A347	*A. longipes*	20.5080	−16.2380	PNBA: Bir el Gareb, 15 km S of	Mauritania
6339	*A. longipes*	19.8079	−16.1479	PNBA: Elb en Nouçç, extreme S	Mauritania
6340	*A. longipes*	19.8079	−16.1479	PNBA: Elb en Nouçç, extreme S	Mauritania
6348	*A. longipes*	19.7819	−16.1880	PNBA: Grâret Agoueifa	Mauritania
6349	*A. longipes*	19.7819	−16.1880	PNBA: Grâret Agoueifa	Mauritania
6414	*A. longipes*	20.5046	−16.3389	PNBA: Îmgoûtene, 5 km NE of	Mauritania
3607	*A. longipes*	19.8232	−16.2100	PNBA: Iouîk, 16 km SE of	Mauritania
6451	*A. longipes*	20.8233	−16.5882	PNBA: Kerekchet et Teintâne, central	Mauritania
6452	*A. longipes*	20.8233	−16.5882	PNBA: Kerekchet et Teintâne, central	Mauritania
5168	*A. longipes*	20.7328	−16.6021	PNBA: Kerekchet et Teintâne, central 1	Mauritania
5163	*A. longipes*	20.7538	−16.5820	PNBA: Kerekchet et Teintâne, E side 1	Mauritania
5164	*A. longipes*	20.7538	−16.5820	PNBA: Kerekchet et Teintâne, E side 1	Mauritania
5167	*A. longipes*	20.7309	−16.5902	PNBA: Kerekchet et Teintâne, E side 2	Mauritania
6438	*A. longipes*	20.6815	−16.5913	PNBA: Kerekchet et Teintâne, extreme S	Mauritania
5177	*A. longipes*	20.7620	−16.6183	PNBA: Kerekchet et Teintâne, W side 3	Mauritania
6317	*A. longipes*	19.6522	−16.2803	PNBA: Kôra	Mauritania
6318	*A. longipes*	19.6522	−16.2803	PNBA: Kôra	Mauritania
2746	*A. longipes*	20.1281	−16.0893	PNBA: Oued Nouafferd	Mauritania
5137	*A. longipes*	20.1507	−16.1211	PNBA: Oued Nouafferd 1	Mauritania
6374	*A. longipes*	20.0845	−16.1313	PNBA: Oued Nouafferd, 2 km S of	Mauritania
6436	*A. longipes*	20.7938	−16.5462	PNBA: Sebkhet Dbâdeb et Teintâne, W margin	Mauritania
6352	*A. longipes*	19.7942	−16.2101	PNBA: Taguîlâlet Jreik	Mauritania
6356	*A. longipes*	19.8455	−16.2014	PNBA: Taguîlâlet Jreik, 1 km W of	Mauritania
6302	*A. longipes*	19.5863	−16.3268	PNBA: Toueigueret, 1 km SW of	Mauritania
6306	*A. longipes*	19.5842	−16.3514	PNBA: Toueigueret, 2 km SW of	Mauritania
A768	*A. scutellatus*	33.5833	2.9500	Bou Trekfine	Algeria
A787	*A. scutellatus*	22.7666	25.6000	Gilf Kebir	Egypt
A133	*A. scutellatus*	32.8968	12.1536	Jadi Resort; 7 km E of Zuara	Libya
8992	*A. scutellatus*	32.3665	−1.3191	Oued Es Safsaf, dunes above dam	Morocco
A086	*A. scutellatus*	33.9000	8.0489	Tozeur, 7 km W of	Tunisia
5823	*A. taghitensis*	22.8047	−12.3783	Zouérat, 11 km NE of	Mauritania

Coordinates are in decimal degrees (WGS84 projection)

*PNBA* Parc National du Banc d’Arguin

## Results and discussion

MICRO-CHECKER revealed no evidence of allelic dropout or stuttering, and no heterozygote excess was observed. In addition, no loci appeared to be in linkage disequilibrium. Table 3 summarizes occurrence of heterozygote deficiency and suspected null alleles for all loci in all populations in the three target species. While the occurrence of null alleles would limit the use of some of these markers in the affected species, other departures from Hardy–Weinberg equilibrium probably result from pooling several sampling localities in the same “populations” (see above). Additionally, even markers showing such evidences might be adequate to apply in other populations and they are applicable in at least one of these species.

**Table 3:** Observations of heterozygote deficiency and null alleles

	*A. aureus*				*A. longipes*				*A. dumerili/senegalensis*			
	Pop1		Pop2		Pop1		Pop2		Pop1		Pop2	
	Het. Def.	Null alleles	Het. Def.	Null alleles	Het. Def.	Null alleles	Het. Def.	Null alleles	Het. Def.	Null alleles	Het. Def.	Null alleles
Ac4						*		*	–	–	–	–
Ac5									*	*		
Ac6	*	*	*	*	–	–	–	–	*	*	*	*
Ac13										*		*
Ac16	*	*		*					–	–	–	–
Ac19							*	*				
Ac23								*				
Ac31							*	*		*		*
Ac32										*		*
Ac33	*	*	*	*					*	*		
Ac43									*	*		*
Ac45			*	*								

Results are presented for *Acanthodactylus aureus*, *A. dumerili/senegalensis* and *A. longipes.* Significant values after Bonferroni correction are marked with an asterisk. Since the heterozygote deficiency was estimated in GENEPOP while null alleles were assessed in MICROCHECKER, differences in the estimation methods may explain the observed lack of concordance between heterozygote deficiency and null alleles in some cases

–, markers that failed to amplify in a certain species

All loci genotyped for each species were polymorphic (Table 4), except for Ac44 that amplified only for *A. longipes*. The Ac36 was also monomorphic in *A. dumerili/A. senegalensis* tested populations but polymorphism was observed in inland samples of this species (own unpublished data, Lopes, Velo-Antón, Crochet, Brito). The number of alleles per locus varied between 5 and 19 in *A. aureus*, and between 1 and 9 in *A. dumerili/A. senegalensis* and *A. longipes*. Expected and observed heterozygosity varied, respectively, between 0.594–0.893/0.188–1.000 in *A. aureus*, 0.223–0.829/0.154–0.826 in *A. dumerili/A. senegalensis* (ignoring Ac36), and 0.046–0.862/0.048–0.905 in *A. longipes* (ignoring Ac44). Most markers amplified in both *A. scutellatus*/17 loci) and *A. taghitensis* (16 loci).

**Table 4:** Characterization of the 22 microsatellite loci

	*A. aureus*								*A. longipes*								*A. dumerili/senegalensis*								*A. scutellatus*			*A. taghitensis*		
	Pop1			Pop2					Pop1			Pop2					Pop1			Pop2										
	N	He	Ho	N	He	Ho	N alleles	Size range in bp	N	He	Ho	N	He	Ho	N alleles	Size range in bp	N	He	Ho	N	He	Ho	N alleles	Size range in bp	N	N alleles	Size range in bp	N	N alleles	Size range in bp
Ac1	20	0.63	0.70	17	0.70	0.59	5	251–267	14	0.25	0.14	21	0.25	0.24	3	271–279	24	0.22	0.25	18	0.29	0.28	4	267–279	2	2	267–271	1	1	263
Ac4	21	0.88	0.76	16	0.89	1.00	16	230–275	6	0.67	0.00	5	0.74	0.20	5	278–290	–	–	–	–	–	–		–	–	–	–	–	–	–
Ac5	21	0.82	0.90	17	0.89	0.94	13	162–198	14	0.60	0.79	21	0.64	0.71	6	141–162	24	0.79	0.54	19	0.61	0.53	7	159–177	5	7	153–182	1	1	197
Ac6	17	0.72	0.35	16	0.76	0.19	9	121–145	–	–	–	–	–	–	–	–	24	0.75	0.50	17	0.79	0.35	8	115–136	5	5	109–139	1	1	121
Ac8	21	0.82	0.76	5	0.78	1.00	10	201–231	14	0.68	0.71	21	0.77	0.71	7	204–234	24	0.38	0.29	19	0.33	0.32	4	198–210	4	4	204–228	1	2	213–216
Ac9	21	0.87	0.86	17	0.85	0.76	17	190–244	–	–	–	–	–	–	–	–	–	–	–	–	–	–		–	–	–	–	–	–	–
Ac13	21	0.83	0.95	17	0.74	0.71	9	140–179	14	0.72	0.64	21	0.69	0.67	8	140–164	23	0.39	0.17	18	0.45	0.28	4	134–161	5	6	134–164	1	1	155
Ac14	21	0.59	0.52	17	0.66	0.53	12	221–266	14	0.24	0.29	21	0.41	0.38	2	118–121	23	0.59	0.61	19	0.45	0.47	3	221–227	3	3	218–224	1	1	224
Ac16	16	0.75	0.31	17	0.79	0.53	8	101–125	14	0.45	0.43	21	0.63	0.57	5	92–113	–	–	–	–	–	–		–	4	4	95–110	–	–	–
Ac19	19	0.87	0.79	6	0.79	0.67	13	208–244	10	0.54	0.40	15	0.72	0.33	6	211–226	24	0.40	0.33	19	0.39	0.42	4	208–217	3	3	214–226	1	1	211
Ac20	17	0.87	0.82	12	0.85	0.67	14	150–201	14	0.65	0.79	21	0.71	0.81	5	168–180	23	0.74	0.83	19	0.76	0.79	9	169–186	1	1	168	1	1	171
Ac23	21	0.67	0.57	17	0.82	0.82	10	116–146	14	0.76	0.64	20	0.76	0.55	7	114–138	24	0.73	0.71	19	0.74	0.68	8	114–135	5	4	123–132	1	2	120–126
Ac31	16	0.80	0.69	17	0.76	0.76	14	306–366	12	0.67	0.58	18	0.59	0.22	7	312–333	23	0.69	0.52	18	0.78	0.56	7	309–327	3	4	321–339	1	2	321–330
Ac32	21	0.85	0.81	15	0.86	0.87	13	232–277	13	0.77	0.77	21	0.86	0.90	9	245–269	20	0.82	0.65	17	0.69	0.41	8	253–274	4	4	254–272	1	2	254–260
Ac33	21	0.83	0.52	16	0.86	0.63	15	120–165	14	0.70	0.50	16	0.63	0.56	5	129–153	23	0.55	0.39	16	0.66	0.50	4	132–144	5	4	132–141	1	2	129–138
Ac36	21	0.84	0.86	16	0.84	0.81	12	110–152	14	0.50	0.50	21	0.36	0.38	2	113–116	23	0.00	0.00	18	0.00	0.00	1	107	4	4	110–119	1	2	128–137
Ac43	18	0.81	0.78	17	0.83	0.71	10	94–124	12	0.61	0.58	21	0.80	0.76	8	106–130	24	0.83	0.50	15	0.82	0.53	9	103–127	4	4	106–126	–	–	–
Ac44	–	–	–	–	–	–	–	–	10	0.00	0.00	20	0.00	0.00	1	202	–	–	–	–	–	–	–	–	–	–	–	–	–	–
Ac45	19	0.85	0.95	17	0.68	0.47	12	133–172	12	0.00	0.00	21	0.05	0.05	2	136–157	24	0.62	0.75	19	0.75	0.74	7	136–157	5	6	136–166	1	2	136–163
Ac47	16	0.83	0.75	17	0.80	0.71	19	187–262	11	0.00	0.00	20	0.14	0.15	2	193–196	–	–	–	–	–	–	–	–	–	–	–	1	1	201
Ac49	17	0.81	0.65	17	0.79	0.71	14	186–225	–	–	–	–	–	–	–	–	–	–	–	–	–	–	–	–	1	1	198	1	1	201
Ac28	16	0.72	0.81	17	0.80	0.65	9	127–166	–	–	–	–	–	–	–	–	–	–	–	–	–	–	–	–	–	–	–	–	–	–
Mean	20	0.82	0.76	17	0.80	0.71	12		14	0.61	0.5	21	0.64	0.47	5		24	0.62	0.5	18	0.66	0.47	7		4	4		1	1	

Sample size (N), number of alleles, allelic size range (expressed in base pairs), expected heterozygosity (He), and observed heterozygosity (Ho) are indicated for *Acanthodactylus aureus, A. dumerili/A. senegalensis* and *A. longipes*. Sample size, number of alleles, and allelic range are also presented for *A. scutellatus* and *A. taghitensis*

–, markers that failed to amplify in a certain species

Although the applicability of each marker may depend on the species considered, the information provided in our work allows a selection of good markers for future use on assessments of genetic structure, genetic diversity, gene flow, and demographic inferences, expanding the possible themes for evolutionary, behavioural and conservation studies in this species group.

## References

[1] Salvador A (1982). A revision of the lizards of the genus *Acanthodactylus* (Sauria: Lacertidae). Bon Zool Monog.

[2] Arnold EN (1983). Osteology, genitalia and the relationships of *Acanthodactylus* (Reptilia: Lacertidae). Bull Brit Mus Nat Hist (Zoology).

[3] Crochet PA, Geniez P, Ineich I (2003). A multivariate analysis of the fringe-toed lizards of the *Acanthodactylus scutellatus* group (Squamata: Lacertidae): systematic and biogeographical implications. Zool J Linnean Soc.

[4] Harris D, Arnold E (2000). Elucidation of the relationships of spiny-footed lizards, *Acanthodactylus* spp. (Reptilia: Lacertidae) using mitochondrial DNA sequence, with comments on their biogeography and evolution. J Zool.

[5] Bons J (1959). Les lacertiliens du Sud-Ouest Marocain: systématique, répartition géographique, éthologie, écologie). Trav Inst Sci Chérifien.

[6] Mellado J, Olmedo G (1990). El género *Acanthodactylus* en Marruecos: problemas de identificación en los grupos de especies *A. pardalis* y *A. scutellatus*. Amphib Reptilia.

[7] Baha El Din SM (1994). A contribution to the herpetology of Sinai. Brit Herp Soc Bul.

[8] Baha El Din SM (2007). A new lizard of the *Acanthodactylus scutellatus* group (Squamata: Lacertidae) from Egypt. Zool Middle East..

[9] Sindaco R, Jeremcenko VK. The reptiles of the Western Palearctic: annotated checklist and distributional atlas of the turtles, crocodiles, amphisbaenians and lizards of Europe, North Africa, Middle East and Central Asia. Latina: Edizioni Belvedere, Monografie della Societas Herpetologica Italica; 2008.

[10] Schlotterer C (2004). The evolution of molecular markers: just a matter of fashion?. Nat Rev Genet.

[11] Wan QH, Wu H, Fujihara T, Fang SG (2004). Which genetic marker for which conservation genetics issue?. Electrophoresis.

[12] Selkoe KA, Toonen RJ (2006). Microsatellites for ecologists: a practical guide to using and evaluating microsatellite markers. Ecol Lett.

[13] Malausa T, Gilles A, Meglecz E, Blanquart H, Duthoy S, Costedoat C (2011). High-throughput microsatellite isolation through 454 GS-FLX titanium pyrosequencing of enriched DNA libraries. Mol Ecol Res.

[14] Schuelke M (2000). An economic method for the fluorescent labelling of PCR fragments. Nat Biotech.

[15] Van Oosterhout C, Hutchinson WF, Wills DP, Shipley P (2004). MICRO-CHECKER: software for identifying and correcting genotyping errors in microsatellite data. Mol Ecol Notes.

[16] Peakall R, Smouse PE (2012). GenAlEx 6.5: genetic analysis in Excel. Population genetic software for teaching and research—an update. Bioinformatics.

